# Camera-Derived Photoplethysmography (rPPG) and Speckle Plethysmography (rSPG): Comparing Reflective and Transmissive Mode at Various Integration Times Using LEDs and Lasers

**DOI:** 10.3390/s22166059

**Published:** 2022-08-13

**Authors:** Jorge Herranz Olazábal, Fokko Wieringa, Evelien Hermeling, Chris Van Hoof

**Affiliations:** 1 IMEC, 3000 Leuven, Belgium; 2 Faculty of Engineering Science, Katholieke Universiteit Leuven (KUL), 3000 Leuven, Belgium; 3 IMEC NL, 5656 AE Eindhoven, The Netherlands; 4 Division of Internal Medicine, Department of Nephrology, University Medical Center Utrecht, 3584 CX Utrecht, The Netherlands

**Keywords:** camera-based, speckle contrast analysis, optical monitoring, laser speckle, PPG, SPG

## Abstract

Background: Although both speckle plethysmography (SPG) and photoplethysmography (PPG) examine pulsatile changes in the vasculature using opto-electronics, PPG has a long history, whereas SPG is relatively new and less explored. The aim of this study was to compare the effects of integration time and light-source coherence on signal quality and waveform morphology for reflective and transmissive rSPG and rPPG. Methods: (A) Using time-domain multiplexing, we illuminated 10 human index fingers with pulsed lasers versus LEDs (both at 639 and 850 nm), in transmissive versus reflective mode. A synchronized camera (Basler acA2000-340 km, 25 cm distance, 200 fps) captured and demultiplexed four video channels (50 fps/channel) in four stages defined by illumination mode. From all video channels, we derived rPPG and rSPG, and applied a signal quality index (SQI, scale: Good > 0.95; Medium 0.95–0.85; Low 0.85–0.8; Negligible < 0.8); (B) For transmission videos only, we additionally calculated the intensity threshold area (ITA), as the area of the imaging exceeding a certain intensity value and used linear regression analysis to understand unexpected similarities between rPPG and rSPG. Results: All mean SQI-values. Reflective mode: Laser-rSPG > 0.965, LED-rSPG < 0.78, rPPG < 0.845. Transmissive mode: 0.853–0.989 for rSPG and rPPG at all illumination settings. Coherent mode: Reflective rSPG > 0.951, reflective rPPG < 0.740, transmissive rSPG and rPPG 0.990–0.898. Incoherent mode: Reflective all <0.798 and transmissive all 0.92–0.987. Linear regressions revealed similar R^2^ values of rPPG with rSPG (R^2^ = 0.99) and ITA (R^2^ = 0.98); Discussion: Laser-rSPG and LED-rPPG produced different waveforms in reflection, but not in transmission. We created the concept of ITA to investigate this behavior. Conclusions: Reflective Laser-SPG truly originated from coherence. Transmissive Laser-rSPG showed a loss of speckles, accompanied by waveform changes towards rPPG. Diffuse spatial intensity modulation polluted spatial-mode SPG.

## 1. Introduction

Photoplethysmography (PPG) is a medical monitoring technology widely used around the globe. PPG opto-electronically records the light intensity modulation scattered and absorbed by living tissue, via a simple light source and light-sensitive detector. The PPG pulse wave is formed by periodic variations in tissue blood volume (caused by heartbeat and respiration) that induce light intensity variations at the photodetector level.

PPG was first recorded on the human ear in 1935 by Matthes, who was not so much interested in the PPG pulse wave, but rather in the much slower oxygen-dependent level shifts in optical absorption from hemoglobin [1]. In 1937, Hertzman built a transmissive mode skin contact probe from a car headlight and a selenium photocell and applied it to fingers and toes [2]. Skin contact probes are still most widely applied (nowadays typically using LEDs and photodiodes). Matthes in 1935 was primarily interested in oxygenation, whereas Hertzman focused upon using PPG pulse waves as a replacement for electromechanical plethysmography (using mercury-impregnated stretchable resistors) as an indicator of skin perfusion. In that context, he perceived the PPG level shifts with varying oxygenation as a “disturbance of the signal” [3]. In 1974, Aoyagi combined the best of both worlds by inventing the pulse oximeter, which derives arterial blood oxygenation from PPG signals simultaneously recorded at two distinct narrow-banded wavelengths [4]. Ever since then, millions of skin-contact probes for placement on fingers, toes, ear lobes or the forehead have been used around the world.

Since 2005, camera-based remote PPG (rPPG) has also become possible [5]. However, when measuring rPPG, the signal-to-noise ratio (SNR) of the signal is much lower in comparison with skin contact probes, and it requires extensive processing to extract a usable rPPG signal from a camera.

Speckle plethysmography (SPG) is much newer than PPG. In 2018, Ghijsen et al. described SPG for the first time [6]. SPG utilizes coherent light, and it is measured by recording the variation in an objective speckle pattern resulting from laser light projected onto tissue. The speckle pattern arises from constructive and destructive photonic interferences as the laser light is scattered by the tissue in transmission or reflection. Imaging detectors enable the recording of the speckle pattern variation that occurs when cells that move in the light-path cause photon path-length variations.

An objective speckle pattern forms when coherent light is projected onto a surface. At the surface level, the irradiated in-phase photons may interact with each other, producing constructive and destructive interferences, which are seen as patterns of bright and dark dots. When the light-source and target volume are static, the speckle pattern remains fixed. However, when particles move within the target volume, the speckle pattern will be modulated.

When capturing a speckle pattern with a camera, the speckle pattern modulation will become more blurred at longer integration times, because light is integrated over time.

This development is called laser speckle contrast imaging (LSCI), and it comes from the speckle theory [7]. LSCI can be applied to hemodynamic measurements because the variation in the contrast of the speckle pattern is related to blood movement and other tissue deformations (respiration/pressure induced), when targeting human tissue.

Laser speckle contrast imaging applied to human measurements is a relatively young area and the differences with the gold-standard biophotonic technology, PPG, have not yet been extensively explored [8,9,10,11,12]. In contrast to PPG, which is dominated by the changes in absorption (with blood hemoglobin as the main chromophore), SPG is dominated by changes in photon pathlength, induced by the movement of cells within the light-path, which, in turn, causes the spatial pulsation of speckle patterns. Given the above differences, we would expect that PPG and SPG contain different information (at least partly), and likely react differently upon variations in different parameters:

- Transmissive versus reflective illumination: Both PPG and SPG can be acquired in diffuse transmissive mode or in diffuse reflective mode. Transmissive mode is limited to applications where the tissue is not too thick and easily accessible from two opposite sides (typically earlobes, fingers, and toes). Reflection mode only requires access from one side, and thus is applicable on far more anatomical sites. For PPG, it is known that transmissive mode generally offers a better signal-to-noise ratio (SNR) than reflective mode. Whether this is also the case for SPG, has (to the best of our knowledge) not yet been thoroughly established.
- Wavelength dependency: Pulse oximetry exploits the differences in spectral absorbance of reduced hemoglobin (Hb) and oxygenated hemoglobin (HbO_2_) to determine SpO_2_ from the AC/DC amplitude ratios of PPGs captured at two different wavelengths (usually chosen in the red and near-infrared ranges). The SPG signal originates from the constructive and destructive interference of coherent light, and thus mainly reacts upon phenomena that change the light pathlength. Scattered photons can still interfere with each other, whereas absorbed photons cannot. Thus, a different wavelength-dependent behavior would be expected between PPG and SPG.
- Coherent versus incoherent illumination: Coherent illumination (laser light) is rarely used for PPG purposes, because the interference speckles contribute to the system noise within the amplitude domain. Hence, PPG is typically acquired using incoherent light (usually LEDs). SPG, however, requires coherent illumination to generate speckle patterns. Hence, lasers are used for SPG applications.

In this study, we investigated the differences between rPPG and rSPG signals—derived from the same video recording via distinct processing methods—regarding signal quality and waveform morphology. We investigated the effects of coherence versus incoherence, transmissive mode versus reflective mode, wavelength (639 nm versus 850 nm), and integration time (600–1400 μs).

## 2. Methods and Materials

We built a camera-based setup, capable of capturing laser speckle contrast images at 200 fps. We applied time-division multiplexing to pulse specific lasers or LEDs in a repetitive cycle, thus dividing the 200 fps from the camera into 4 video-channels of 50 Hz each). We also 3D-printed 2 custom light source holders: one for reflective illumination, and one for transmissive illumination (in the form of a hand rest to comfortably support the index finger). Both light source holders contained a 639 nm laser, an 850 nm laser, a 639 nm LED, and an 850 nm LED.

We designed and built a system to record the camera signals, synchronously triggered alongside the Laser and LED illumination sources of 639 nm and 850 nm, as multiplexed in time. We designed 4 illumination modes with 4 multiplexed lights each, to investigate the effect in signal quality of coherence, light-paths, and integration time. These modes were: transmissive, reflective, coherent, and non-coherent, and each mode consisted of 4 light sources.

The setup was used to illuminate the index finger of 10 healthy human volunteers. We processed the recorded video streams to calculate intensity modulations and spatial variations in the captured speckle patterns, producing both rPPG and rSPG signals from each of type of illumination. The experimental protocol was evaluated and executed in accordance with to the Declaration of Helsinki 2008 (protocol number IP-20-WATS-TIP2-085).

During each stage, recordings were made at 5 different camera integration time settings of 600, 800, 1000, 1200, and 1400 µs (each for 1 min duration) to identify the optimum setting to maximize the heart-cycle information on simultaneous single-exposure PPG and SPG in comparison to the noise.

### 2.1. Instrumental Assembly

The setup was built around an area-scan CMOS camera (Basler acA2000-340 km; ROI set to 512 × 320 pixels, 200 fps) placed at 25 cm distance, imaging the index finger from the top. This camera was triggered via a microcontroller (STM32) that also controlled the timing of the applied LEDs and lasers (thereby synchronizing all system components). The laser driver was home-built using an iC-WJZ chip (iC Haus).

The camera was triggered at a framerate of 200 fps. Via time domain multiplexing, the camera framerate was equally distributed across 4 acquisition channels (each 50 fps). This enabled continuous video acquisition while multiplexing between different illumination modes and/or light source types. The following light source types were applied:

- Red laser diode (Thorlabs HL6358MG—639 nm, 10 mW, Ø5.6 mm);
- NIR laser diode (Thorlabs L850P010—850 nm, 10 mW, Ø5.6 mm);
- Red LED (Wurth Elektronik 150141SS63140—640 nm, 196 mW);
- NIR LED (Vishay TSHG6200—850 nm, 180 mW).

**Figure 1:**
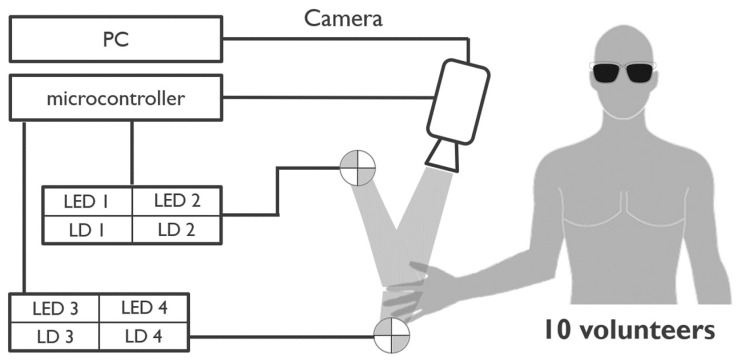
Measurement setup diagram.

The camera images were stored in a desktop computer using full camera-link communication, as detailed in Figure 1.

### 2.2. Experimental Protocol

Table 1 lists the 4 experimental modes with their 4 respective illumination settings.

The experimental protocol was composed of 4 stages:

1. Reflective Mode: 1 red laser diode, 1 NIR laser diode, 2 red LEDs, and 2 NIR LEDs illuminated the finger from the top (using time domain multiplexing). This stage serves to investigate the effect of coherent versus incoherent illumination upon the signal qualities of reflective PPG and reflective SPG upon signal qualities.
2. Transmissive Mode: The same light source types as in reflective mode were used but placed below the finger (facing the distal phalanx from the fingerprint side), thus forcing the photons to transmit through the finger. This stage aims to analyze the effect of coherence in transmissive PPG and transmissive SPG signal qualities.
3. Coherent Mode: Only laser light was applied. Pairs of red and NIR laser diodes were applied both in reflective and transmissive mode. This stage aims to analyze the effect of light-path in rPPG and rSPG signal qualities when only using coherent light.
4. Non-Coherent Mode: Only LED light was applied. Pairs of red and NIR LEDs were applied both in reflective and transmissive mode. This stage aims to analyze the effect of light-path in rPPG and rSPG signal qualities when only using incoherent light.

During each stage, recordings were made at 5 different camera integration time settings of 600, 800, 1000, 1200, and 1400 µs (each for 1 min duration) to identify the optimum setting.

**Figure 2:**
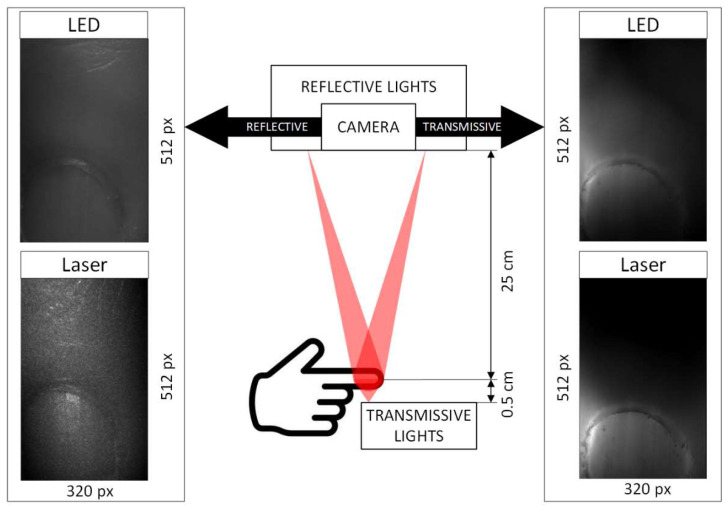
Setup diagram with sample 639 nm raw images in reflective (**left**) and transmissive mode (**right**). Left top: reflective image with LED illumination. Left bottom: reflective image with laser illumination (clear speckle pattern visible). Right top: transmissive image with LED illumination. Right bottom: transmissive image with laser illumination.

All participants signed an informed consent form. They were placed in a sitting position and instructed how to place their finger and wear laser safety goggles. They were given 10 min of rest to allow for physiological stable conditions. All experiments were performed in a dimly lit room. Figure 2 shows example raw images from the system.

### 2.3. Processing of Video Data

#### 2.3.1. Processing of rSPG and rPPG

**Figure 3:**
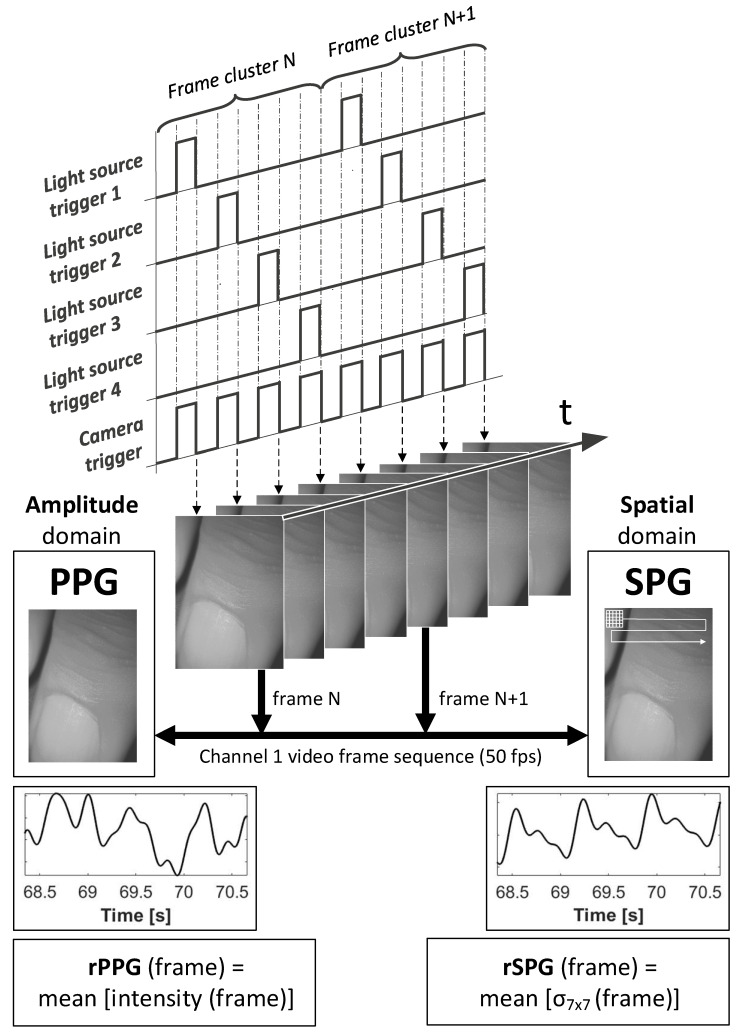
Raw camera video frames are captured at 200 fps and by time-domain-multiplexing split-up into 4 channels, each pulsing a different light source at 50 fps. Two different processing methods—amplitude domain and spatial domain—are both applied to the same video stream. Here, we show rPPG versus rSPG for Channel 1. Note that raw waveforms are displayed vertically inverted, as is common practice for commercial PPG and SPG signal outputs.

The same camera video sequences were processed with two different algorithms to derive pulse waves from absorption modulation (PPG) and from spatial speckle pattern modulation (SPG), see Figure 3.

For rPPG processing, the average intensity frame by frame over time was calculated. We partially based the processing to obtain rSPG upon the method described by Ghijsen et al. [6]. The first step was a standard deviation mask (σ)—sized 7 × 7 pixels, based on the speckle–pixel size ratio—which was scanned across the whole image for each video frame. As a second step, all values resulting from this standard deviation mask-scan were then averaged per video frame. Note: In contrast to Ghijsen et al., we avoided dividing by the mean intensity per frame on the calculation of rSPG to optimally decouple from rPPG and maximize ambient light suppression [13].

#### 2.3.2. Processing of Intensity Threshold Area (ITA)

In transmissive mode, images showed a lack of a speckle pattern; however, the rSPG algorithm gave clear pulsatile results similar to rPPG, probably arising from varying areas of intensity. To enable better interpretation of the similarities of rPPG and rSPG signals in transmissive mode, in each frame in the region with intensity above a predefined threshold value was extracted. The so-called intensity threshold area (ITA) was defined as the number of pixels within this region calculated for every time frame. 

**Figure 4:**
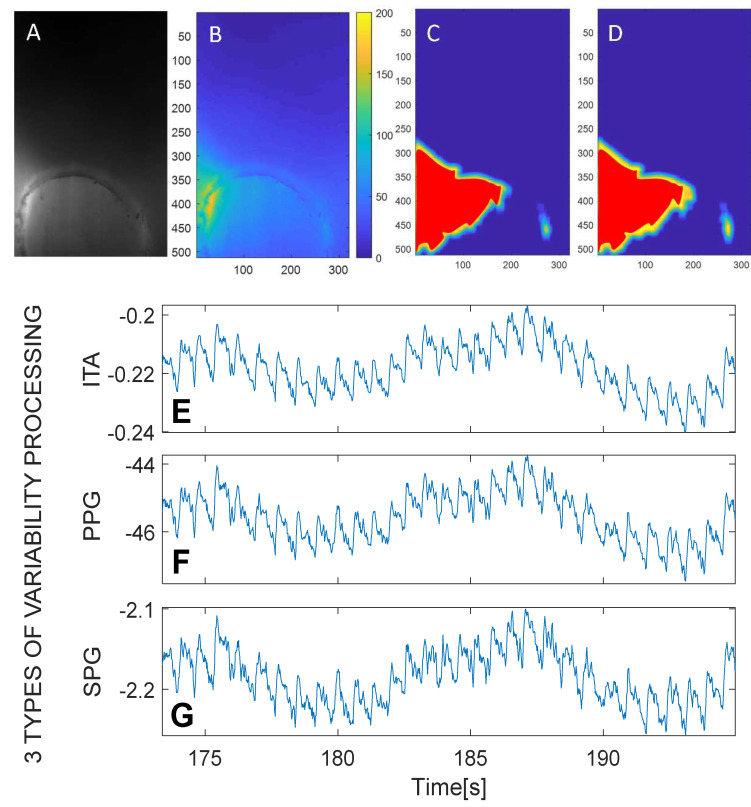
Expansion of the intensity pattern in transmission measurements causing spatial variability changes originated from absorption changes together with rPPG and rSPG. (**A**) Raw video image. (**B**) False color-coded intensity image. (**C**) Systolic (t = 183.1 s) portion of the false color-coded image above a certain intensity threshold. (**D**) Diastolic (t = 183.9 s) portion of the false color-coded image above the same intensity threshold. This area is somewhat expanded compared with C. (**E**) ITA pulsation. (**F**) Transmission rPPG. (**G**) Transmission rSPG. Note that raw waveforms are displayed vertically inverted, as is common practice for commercial PPG and SPG signal outputs. PPG and SPG synchronous signals from a random subject with 1200 μs integration time.

Figure 4C,D depicts the image after thresholding at a certain intensity value. With the intention of making the comparison easier between expanded and contracted stages of ITA, we superimposed a red area which did not vary in surface.

## 3. Analysis

### 3.1. Signal Quality Analysis of rSPG and rPPG

To compare the signal quality of rSPG with rPPG under the different configuration modes, we designed a signal quality index (SQI). The SQI value followed from the ratio of the spectral power density contained in two frequency bands. The first band was the physiological band (0.4–15 Hz), and the power density was calculated through the root mean square (RMS) of the band-passed signal. The second band contained all frequencies from 0.4 Hz onwards, and the power density was calculated through the RMS of the high-passed signal. Dividing the power density of the first band by the second band, gave a signal-to-noise ratio. The filters were designed in MATLAB (Butterworth type, order 12).

$$\mathrm{SQI}=\frac{\mathrm{RMS}\;\left({\mathrm{BP}}_{0.4–15\;\mathrm{Hz}}\;\mathrm{filter}\;\left(\mathrm{Signal}\right)\right)}{\mathrm{RMS}\;\left({\mathrm{HP}}_{0.4\;\mathrm{Hz}}\;\mathrm{filter}\;\left(\mathrm{Signal}\right)\right)}$$

In other words, this ratio provides a surrogate SNR, only intended as an objective indication of the spectral power density of the primary peak and harmonics when comparing rPPG and rSPG signals derived from a shared raw input video stream. The absolute system noise level within the captured video streams will vary with integration time, optical power, finger position, etc., but our study compared the differences between the signals derived by the different processing pipelines with the incoherent/coherent light-sources and transmissive/reflective modes. A disadvantage of this comparison method is that it disregards information from respiration and Mayer Waves.

### 3.2. Regression Analysis of rSPG, rPPG, and ITA

We investigated whether the pulsatile spatial information of iso-intensity lines provided by ITA correlated with the signals produced by both rPPG and rSPG processing in *transmissive mode*.

To determine the similarities, we performed linear regressions between rPPG and rSPG as well as between rPPG and ITA pulsation. We provide a calculation of R-squared, standard error, and the number of observations.

## 4. Results

### 4.1. Results of Signal Quality Analysis between rSPG and rPPG

This section summarizes the results of signal quality analysis, giving graphical examples of the PPG and SPG on the four different stages of the experiments (see Table 2 and Table 3). For visualization purposes, we only plotted signal traces obtained with 1200 µs integration time, but the statistics have been extracted from all parts of the experiment (600 to 1400 µs) for all 10 subjects. We provide the same objective quality index for both PPG and SPG for all subjects.

#### 4.1.1. Reflective Mode Results

This part of the experiment was designed to determine the effect of coherence on the quality of reflective rSPG (R-rSPG) and reflective rPPG (R-rPPG) signals under different integration times. In this stage, we illuminated the finger with four reflective light sources multiplexed in time:

- Lasers and LEDs of 639 nm (reflection);
- Lasers and LEDs of 850 nm (reflection).

**Figure 5:**
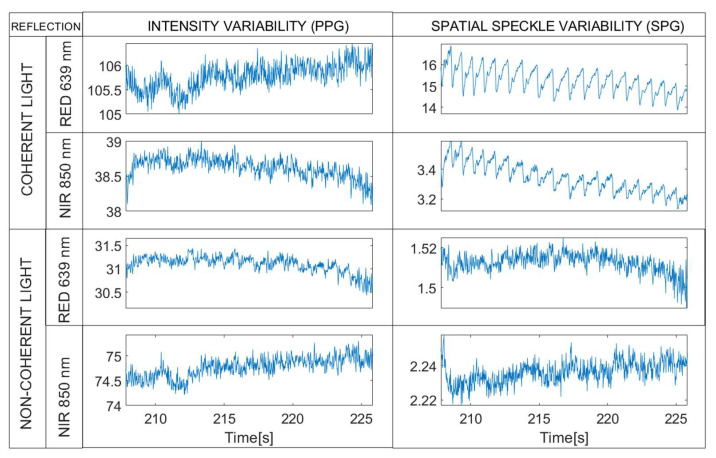
Reflective mode. Raw synchronous R-rPPG (**left**) and R-rPPG (**right**) signals extracted from the 4 multiplexed light sources (1200 µs integration time). As expected, good SPG quality was observed when laser light sources are used. Note that raw waveforms are displayed without vertical inversion—PPG and SPG synchronous signals from a random subject with 1200 µs integration time.

As expected, lasers produced good-quality SPG signals due to the considerable changes in the blurriness of the speckle pattern, which induce considerable changes in the spatial variability in the images. As also expected, LEDs produce SPG signals of very low quality in reflective mode because there is no speckle pattern from which to obtain information. R-rPPG is mostly ruled by specular reflection, giving signals of very low quality with all light-sources (see Figure 5).

**Figure 6:**
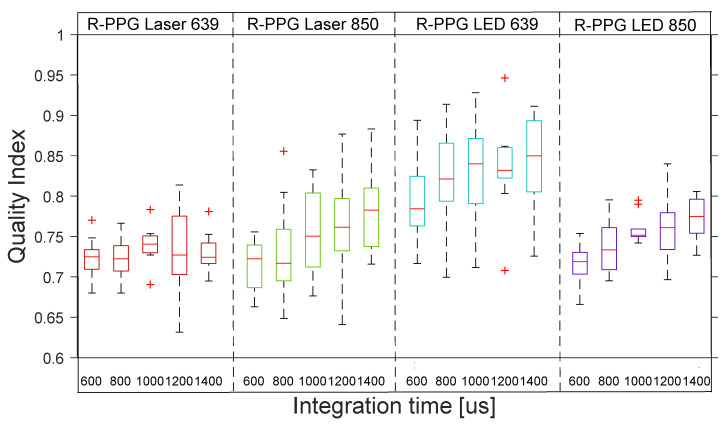
Reflective mode boxplots of quality index for R-rPPG signals with 4 different lights at 5 integration times. The inner box divisions indicate: Q1 (the first quartile) to Q3 (the third quartile). The median is marked by a line across the box. The “whiskers” indicate results from Q1 and Q3 to the most extreme data points. The + and ‡ symbols represent outliers.

Figure 6 shows the distribution across the 10 subjects of the quality indexes for R-rPPG with the 4 reflection lights. The boxplot distribution of the reflective mode shows the low quality of all the R-rPPG signals across the 10 subjects.

**Figure 7:**
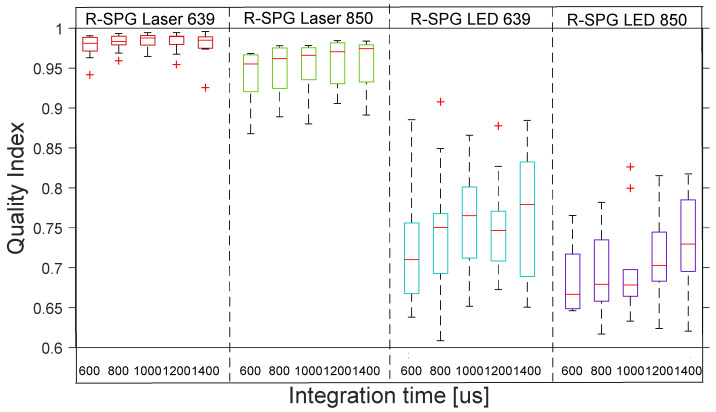
Reflective mode boxplots of quality index for R-rSPG signals with 4 different lights at 5 integration times in the reflection. The inner box divisions indicate: Q1 (the first quartile) to Q3 (the third quartile). The median is marked by a line across the box. The “whiskers” indicate results from Q1 and Q3 to the most extreme data points. The + symbols represent outliers.

Figure 7 shows the distribution across the 10 subjects of the quality indexes for R-rSPG with the 4 reflection lights. The boxplot distribution of the reflective mode shows the high quality of R-rSPG signals produced by lasers and the poor quality of SPG signals produced by LEDs across the 10 subjects.

#### 4.1.2. Transmissive Mode Results

This part of the experiment was designed to investigate the effect of coherence on the quality of transmissive rSPG and rPPG signals under different integration times. In this stage, we illuminated the finger with four transmissive lights multiplexed in time:

- Lasers and LEDs of 639 nm (transmission);
- Lasers and LEDs of 850 nm (transmission).

Lasers and LEDs showed good-quality rPPG, but very unexpectedly also both showed high SQI values for rSPG signals. Oddly, even though LEDs do not produce coherent light, they contribute to the spatial variability in transmissive mode. This is a huge contrast with reflective mode results.

**Figure 8:**
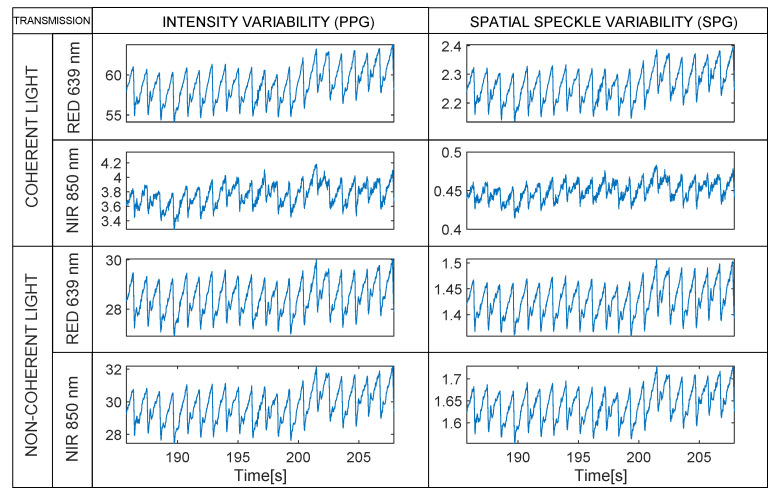
Transmissive mode. Synchronous rPPG (**left**) and rSPG (**right**) signals extracted from the 4 multiplexed light sources. Note the strong similarity in waveform morphology, as opposed to the reflective mode results in Figure 4. Note that raw waveforms are displayed without vertical inversion—PPG and SPG synchronous signals from a random subject with 1200 µs integration time.

Figure 8 shows how the rPPG and rSPG waveforms are almost identical with all light-sources when measured in transmission. A close look at the raw videos revealed that the speckle pattern disappeared in transmission videos with this optical setup; however, we still obtained an “SPG” signal. This observation triggered us to perform an additional analysis (see Section 3.2, Analysis B). In the Discussion, we elaborate an explanation for this phenomenon.

**Figure 9:**
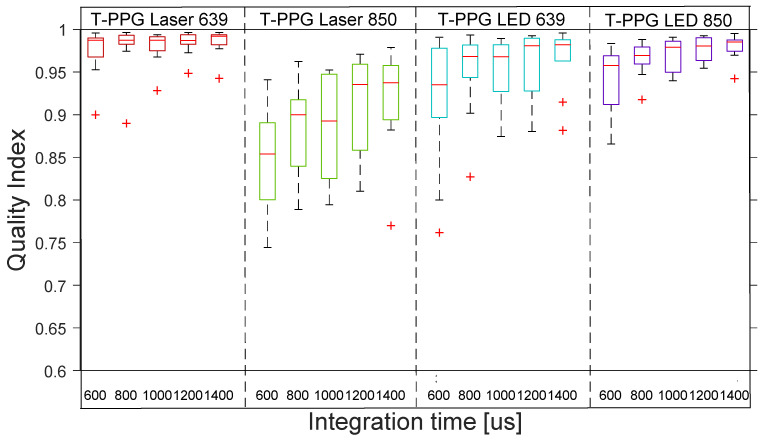
Boxplots of quality index of rPPG signals with 4 different lights at 5 integration times in transmission. The inner box divisions indicate: Q1 (the first quartile) to Q3 (the third quartile). The median is marked by a line across the box. The “whiskers” indicate results from Q1 and Q3 to the most extreme data points. The + symbols represent outliers.

Figure 9 shows the distribution across the 10 subjects of the quality indexes for rPPG with the 4 transmission lights. The boxplot distribution of the transmissive mode shows the high–medium quality of all the rPPG signals across the 10 subjects.

**Figure 10:**
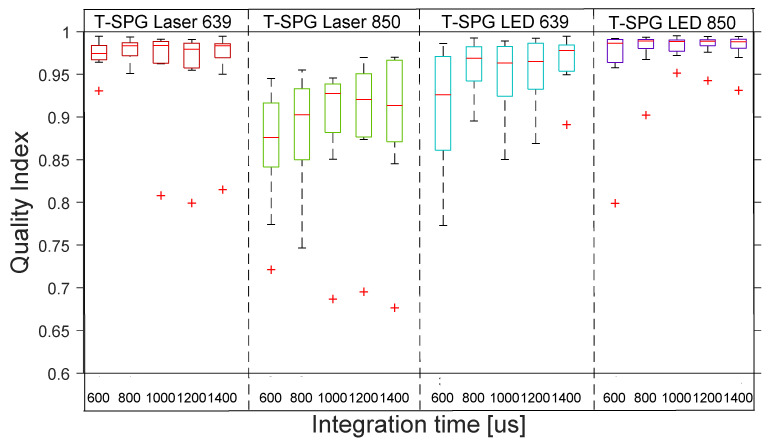
Boxplots of quality index of rSPG signals with 4 different lights at 5 integration times in transmission. Note the higher signal quality of 850 nm “SPG” signal when using LEDs instead of lasers. The inner box divisions indicate: Q1 (the first quartile) to Q3 (the third quartile). The median is marked by a line across the box. The “whiskers” indicate results from Q1 and Q3 to the most extreme data points. The + symbols represent outliers.

Figure 10 shows the distribution across the 10 subjects of the quality indexes for rSPG with the 4 transmission lights. The boxplot distribution of the transmissive mode shows the high–medium quality of all the rSPG signals across the 10 subjects.

#### 4.1.3. Coherent Mode Results

**Figure 11:**
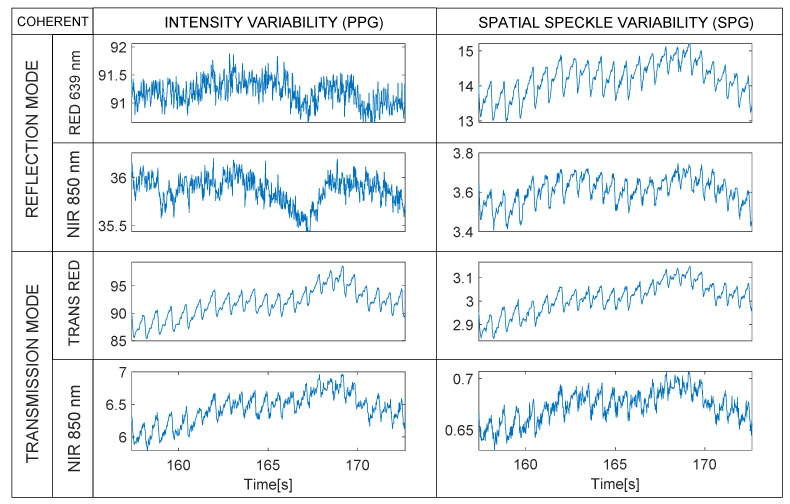
Coherent mode. Synchronous rPPG (**left**) and rSPG (**right**) signals extracted from the 4 multiplexed Laser light sources (1200 µs integration time). Note the differences in waveform morphology in reflective mode, versus the similarities in transmissive mode. Note that raw waveforms are displayed without vertical inversion—PPG and SPG synchronous signals from a random subject with 1200 µs integration time.

This part of the experiment was designed to investigate the effect of light-path on the quality of rSPG and rPPG signals with coherent light illumination under different integration times. In this stage, we illuminated the finger with two transmissive and two reflective laser lights multiplexed in time (see Figure 11):

- Lasers 639 nm and 850 nm (transmission);
- Lasers 639 nm and 850 nm (reflection).

In reflection lasers show negligible R-rPPG and good-quality R-rSPG, whereas in transmission they show medium–good quality for both rSPG and rPPG.

In this stage, we appreciate how the rPPG and rSPG are almost identical when measured in transmission with the current optical setup. Additionally, even having the same periodicity they differ in waveform from the reflection SPG.

The information in reflection R-rSPG comes from the variation in the speckle pattern. However, in transmission, when looking at the images, we noticed the disappearance of the speckle pattern. The rSPG variation was not produced by the speckle pattern, but a different phenomenon. We elaborate an explanation in the Discussion for this phenomenon.

**Figure 12:**
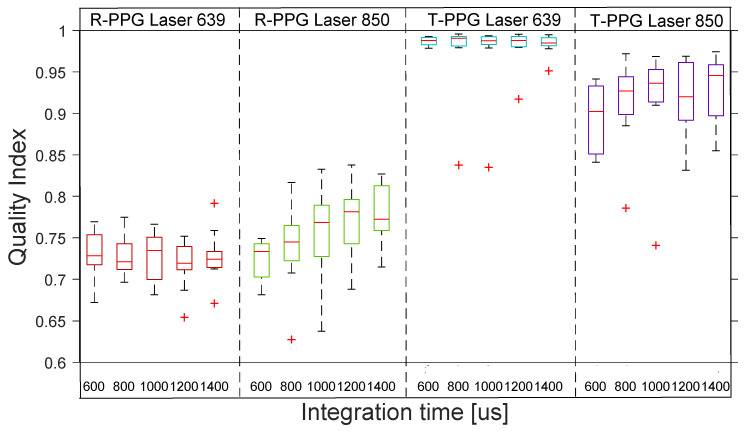
Boxplots of quality index of rPPG signals with 4 different lights at 5 integration times with coherent light in transmission and reflection. The inner box divisions indicate: Q1 (the first quartile) to Q3 (the third quartile). The median is marked by a line across the box. The “whiskers” indicate results from Q1 and Q3 to the most extreme data points. The + symbols represent outliers.

Figure 12 shows the distribution across the 10 subjects of the quality indexes for rPPG with 4 coherent lights in transmission and reflection. The boxplot distribution of the reflective laser shows low-quality rPPG signals across all subjects. The transmissive laser shows the high–medium quality of all the rPPG signals across the 10 subjects.

**Figure 13:**
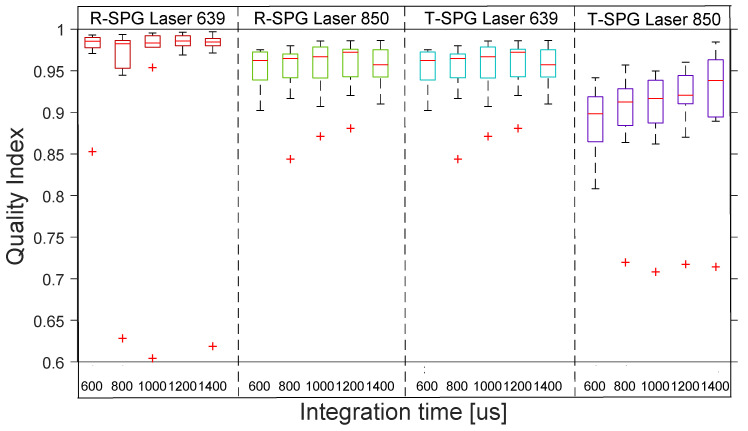
Boxplots of quality index of rSPG signals with 4 different lights at 5 integration times with coherent light in transmission and reflection. The inner box divisions indicate: Q1 (the first quartile) to Q3 (the third quartile). The median is marked by a line across the box. The “whiskers” indicate results from Q1 and Q3 to the most extreme data points. The + symbols represent outliers.

Figure 13 shows the distribution across the 10 subjects of the quality indexes for rSPG with 4 coherent lights in transmission and reflection. The boxplot distribution shows good quality rSPG signals for all laser light sources.

#### 4.1.4. Non-Coherent Mode Results

**Figure 14:**
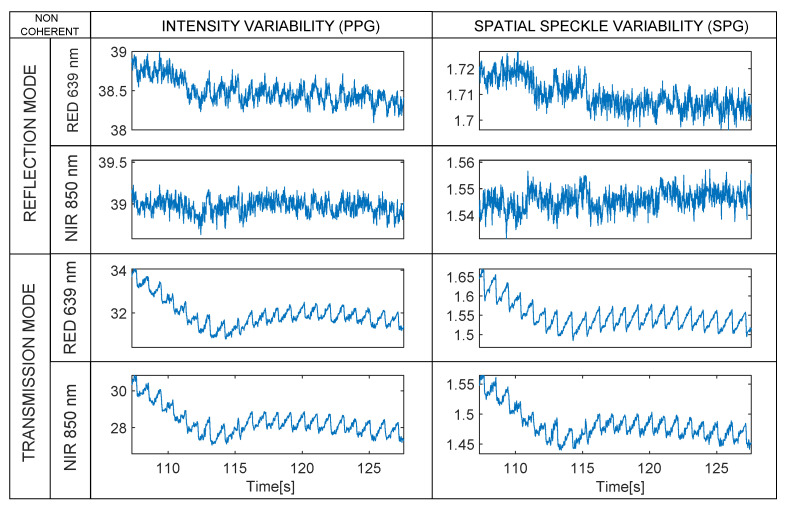
Non-coherent mode. Synchronous rPPG (**left**) and rSPG (**right**) signals extracted from the 4 multiplexed LED light sources (1200 µs integration time). Note that raw waveforms are displayed without vertical inversion—PPG and SPG synchronous signals from a random subject with 1200 µs integration time.

This part of the experiment was designed to investigate the effect of light-path on the quality of rSPG and rPPG signals with non-coherent light illumination under different integration times. In this stage, we illuminated the finger with two transmissive and two reflective LED lights multiplexed in time (see Figure 14):

- LED 639 nm and 850 nm (transmission);
- LED 639 nm and 850 nm (reflection).

In reflection, LEDs induce negligible R-rPPG and R-rSPG, whereas in transmission they exhibit medium–good-quality rSPG and rPPG.

In this stage, we appreciate how the rPPG and rSPG are almost identical when measured in transmission with the current optical setup even with non-coherent light.

In transmission images, there is obviously no speckle pattern to obtain information from, although good-to-medium-quality signals are still produced. The rSPG variation apparently was not produced by the speckle pattern, but a different phenomenon. We elaborate an explanation in the Discussion for this phenomenon.

**Figure 15:**
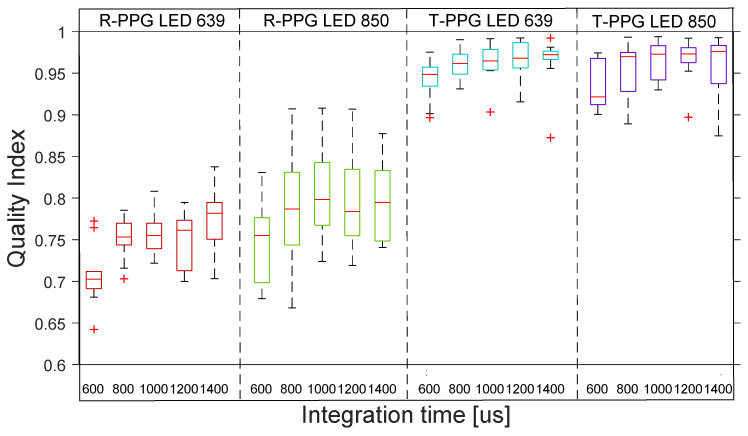
Boxplots of quality index of rPPG signals with 4 different lights at 5 integration times with non-coherent light in transmission and reflection. The inner box divisions indicate: Q1 (the first quartile) to Q3 (the third quartile). The median is marked by a line across the box. The “whiskers” indicate results from Q1 and Q3 to the most extreme data points. The + symbols represent outliers.

Figure 15 shows the distribution across the 10 subjects of the quality indexes for rPPG with 4 non-coherent lights in transmission and reflection. The boxplot distribution of the reflective LEDs shows low-quality rPPG signals across all subjects. The boxplot distribution of transmissive LEDs shows the high–medium quality of all the rPPG signals across the 10 subjects.

**Figure 16:**
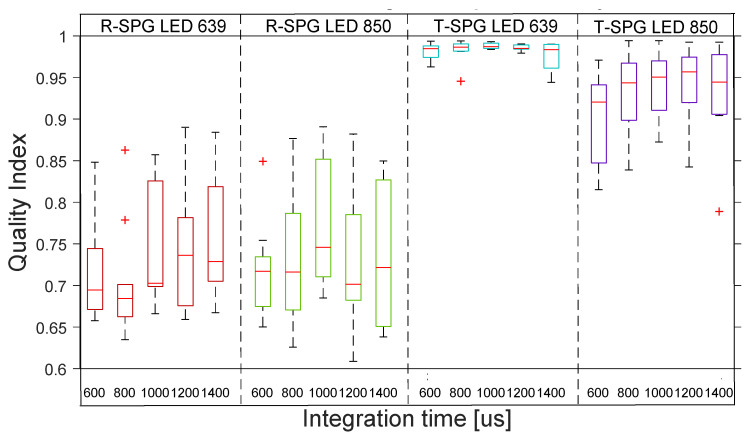
Boxplots of quality index of rSPG signals with 4 different lights at 5 integration times with non-coherent light in transmission and reflection. The inner box divisions indicate: Q1 (the first quartile) to Q3 (the third quartile). The median is marked by a line across the box. The “whiskers” indicate results from Q1 and Q3 to the most extreme data points. The + symbols represent outliers.

Figure 16 shows the distribution across the 10 subjects of the quality indexes for rSPG with 4 non-coherent lights in transmission and reflection. The boxplot distribution shows bad-quality rSPG signals for reflective LED light sources. The boxplot distribution shows good-quality rSPG signals for transmissive LED light sources.

### 4.2. Results of Regression Analysis between rPPG and ITA and rSPG

**Figure 17:**
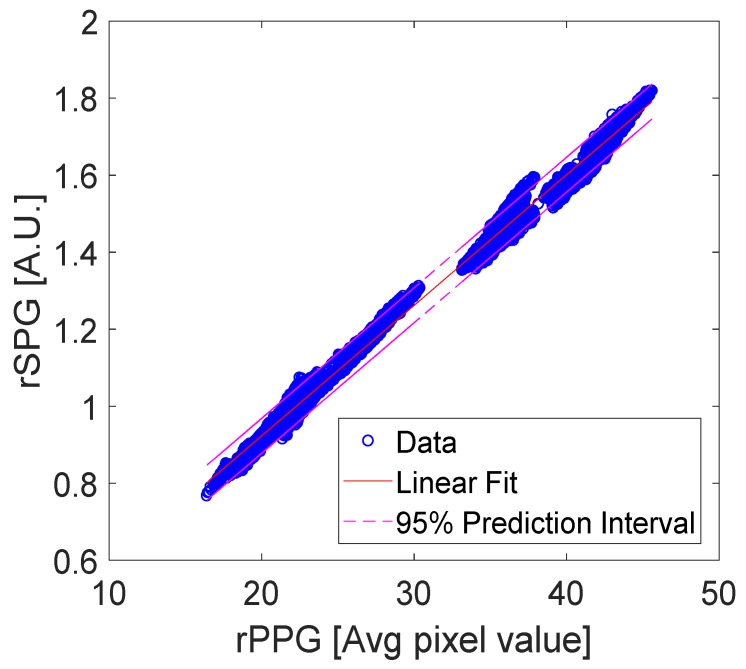
Linear regression of rPPG and rSPG data with a 95% prediction interval.

**Figure 18:**
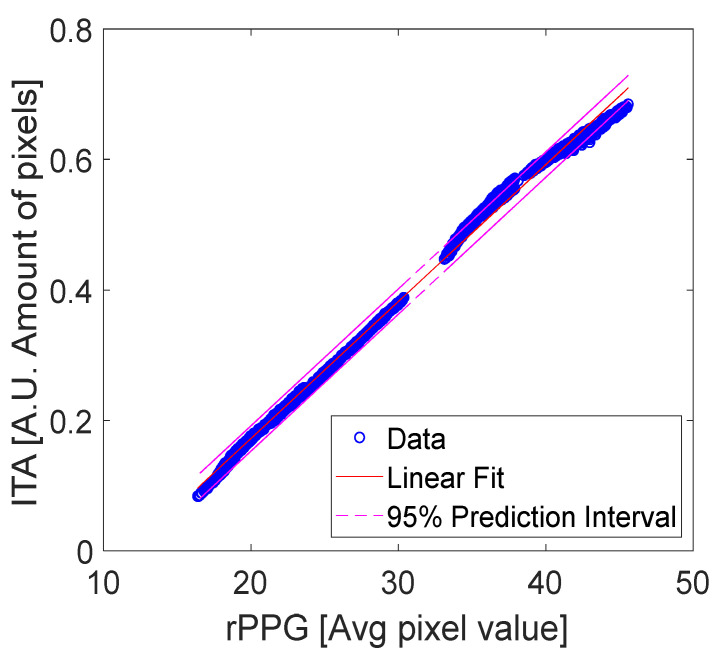
Linear regression of rPPG and ITA data with a 95% prediction interval.

Regression tests (using Matlab’s Polyfit and Polyval) are shown for rPPG vs. rSPG signals (Figure 17 and Table 4), and rPPG vs. ITA signals (Figure 18 and Table 5) for all integration times. The input data used for these tests were synchronous because the three signals (rPPG, rSPG, and ITA) were always produced from the same video streams. We performed the test on unfiltered signals. Regression tests on rPPG vs. rSPG showed a slightly higher R^2^ = 0.99 than that of PPG vs. ITA (R^2^ = 0.98).

## 5. Discussion

To the best of our knowledge, this is the first time that the effect of transmission versus reflection, wavelength, coherence, and integration time on rPPG and rSPG signal quality have been examined. The results show that rSPG has superior qualities over rPPG in reflection mode, whereas in transmission mode, rPPG and rSPG have very similar patterns. Results also showed that the best-quality rSPG, without showing pollution from rPPG, was obtained with coherent light in reflective mode.

### 5.1. Signal Quality Index

We needed an objective means to evaluate signal qualities of both our PPG and SPG pipelines; therefore, we first looked for previous publications comparing PPG and SPG signal qualities from the same device. We only found one reference that compared the quality of PPG and SPG from the same camera-based images, namely, the work of Dunn et al. [11]. They, however, used a rudimentary signal quality comparison, namely, by only comparing the amplitude of the fundamental heart rate frequency after dividing by the DC component. Hence, we thought that it would be an improvement to not divide by the DC component (because it does not represent the same in PPG and SPG) and, in addition to the fundamental heart frequency, also includes other frequency bands containing useful harmonics.

**Figure 19:**
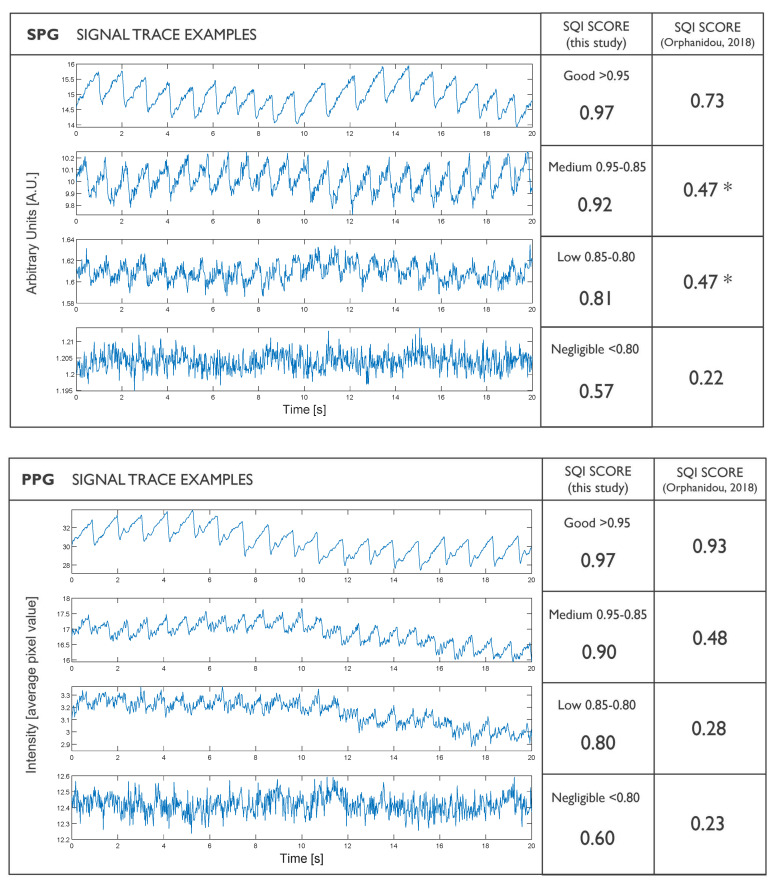
Comparison between SQI scales used in this study and a sophisticated widely used SQI template specifically developed for PPG. The template PPG SQI works better for PPG, although it does not work well for SPG. * Note the SPG values marked with an asterisk, which score as medium and low with our SQI scale, whereas both scored the same 0.47 value with the template SQI method.

We evaluated the SQI used in this article by comparing PPG and SPG signals of different qualities using our SQI versus using a template-standardized SQI algorithm, such as that published by Allen and Kyriacou [14], reacting upon the work of Orphanidou [15]. For PPG, this reference algorithm performs better than ours, but with SPG signals it performs worse (this is logical because that algorithm was not tailored to SPG). Our SQI algorithm provides a usable scale for both PPG and SPG by comparing the ratio between signal content and system noise. It is certainly not the ultimate solution, but it is meaningful to objectively compare PPG and SPG signals obtained from the same device (see Figure 19).

### 5.2. Transmission versus Reflection

**Reflection**: Measurements in reflection with coherent light showed heart-rate components of very good quality in the spatial variability domain (rSPG). However, intensity processing of the same videos showed negligible heart-rate components in the intensity domain (rPPG). With the same reflective illumination conditions, but non-coherent light, the measurements showed negligible heart-rate components in both domains. Only the NIR LED shows a smooth HR component in the intensity domain, but it is likely not sufficient to calculate HRV.

**Transmission**: Measurements in transmissive mode showed high SQI values for both the intensity (rPPG) and the spatial variability domain (rSPG), but surprisingly regardless of whether coherent or incoherent light sources were used. Moreover, intensity and variability domain processing showed nearly identical waveform morphologies. This was unexpected, because in a previous study we observed that reflective rSPG pulses showed significantly sharper edges than transmissive finger probe PPG [13]. Triggered by this unexpected behavior, we then visually analyzed the transmission videos and noticed that in transmissive mode, the speckle pattern disappeared. We reasoned that the cause may be the photons reaching the detector, which have been scattered inside the tissue, which has a volumetric flow, show a loss of coherence, which makes it less likely to form interference speckle patterns, or may result in much smaller speckles. With stronger magnification lenses or other types of lasers, it might still be possible to obtain speckle patterns in transmissive mode (but this warrants further investigations). We did not have the possibility to directly measure laser coherence length and only had a laser output spectrum for our NIR laser (850 nm L850P010). Therefore, we used an approximation:

(1) $$L_{c}\approx \frac{\lambda^{2}}{∆\lambda }=\frac{850^{2}}{0.8}\left[\mathrm{nm}\right]=0.903\;\left[\mathrm{mm}\right]$$

With a full width at half maximum value (Δλ) of 0.8 nm, the according NIR coherence length was 0.9 mm. This approximation matched with typical values found in free running laser diodes [16]. For our red laser (639 nm HL6358MG), we unfortunately did not have access to a laser spectrum or direct coherence length specifications. However, even if the red laser were to have a fivefold longer coherence length, this stays well below the thickness of any human adult finger. It is thus reasonable to assume that in transmission mode, coherence is largely lost. In reflection mode, much more back-scattered photons will have a chance to stay within the coherence length. This matches with our observations in the raw reflective and transmissive images.

These two observations indicated that the pulsatile rSPG information in transmission measurements (at least with the optical arrangement used in this study) in fact might not originate from the variability in speckle contrast. This led us to develop a second processing method named ITA.

To analyze the similarity of rSPG and rPPG with the results from ITA processing, we performed two linear regression tests: the first one compares rPPG with rSPG, and the second compares rPPG with ITA pulsation. Results of these regression tests revealed 99% and 98% coincidence of the rPPG and rSPG signals with ITA pulsation, respectively.

We can conclude that rPPG absorption changes in transmission, becoming dominant even in the spatial variability domain for transmission measurements.

In transmission, measurements with non-coherent light show the same behavior as with coherent, decent quality rSPG and rPPG signals. This demonstration explains the obtention of decent quality rSPG signals even when not illuminating with coherent light. This proves that during transmission measurements, the rSPG algorithm can be polluted by information not coming from speckle, but from intensity.

### 5.3. Coherent versus Non-Coherent

**Coherent**: Measurements with coherent light in reflection showed signals of very good quality in the spatial variability domain and absence of a heart-rate component in the intensity domain signals

Transmission measurements showed signals with very similar morphology in the spatial and intensity domains. As proven above, in transmission measurements, speckle disappeared from the video and the spatial and intensity variability were ruled by volume changes (rPPG). This resulted in similar rSPG and rPPG signals. It is important to address that simultaneous transmission and reflection rSPG signals differ in waveform.

**Non-Coherent**: Measurements in reflections with non-coherent light produced negligible signals in the spatial variability domain. In conclusion, reflection rSPG does not work with non-coherent light.

Measurements in transmission showed, as expected, very similar signals in spatial and intensity domains. Again, transmission and reflection rSPG morphologies differ. As confirmed by the experiments above, in transmissive mode, spatial variations are formed by an expanding and contracting light distribution, which is ruled by volume changes (rPPG).

Spatial and intensity variability processing showed signals of almost identical morphology, although there was no coherence. This supports the hypothesis that in transmissive mode, with current optical arrangement, the spatial variability is ruled by volume changes (rPPG).

### 5.4. Effects of Integration Time: Finding the Optimum

Boxplot distributions indicated that integration time is significantly beneficial for rPPG signal quality. However, the SPG signal showed a non-significant quality improvement, reaching its maximum SQI values at 1200 µs and then decreasing with longer integration times. This was analyzed from 600 µs to 1400 µs, due to the speed of blood; if longer integration times are utilized, a decrease in SNR is expected, due to excessive blurriness of the speckle pattern.

In contrast to this and giving more strength to the proven hypothesis on the origin of the spatial variation in transmission measurements, all transmission measurements showed clear benefit from the increase in integration time. This can be explained because the information stops coming from the blurriness of the speckle pattern and more integration time means more photons that have been scattered inside the tissue, which means more SNR.

**Increase in integration time affected the quality index results, indicating:**

In reflective mode, for coherent light in the intensity domain, the increase in integration time is beneficial in a significant way. However, in the spatial variability domain, there is no significant advantage beyond 1200 µs. In reflective mode for non-coherent light, the increase in integration time is beneficial for both intensity and spatial domains.

In transmissive mode, for coherent, as for non-coherent light, in both the intensity and spatial variability domains, the increase in integration time is beneficial in a significant way.

The increase in integration time brings more information for rPPG, but should decrease the amount of information on rSPG at a certain point; this behavior is seen in reflective mode. However, in transmission measurements, the spatial variability is a measurement of the changes in iso-intensity lines, which increases the amount of information with longer integration times. This is another indication of the fact that rSPG transforms into rPPG in transmissive mode. Figures with all integration times applied can be found in Supplementary Materials.

The exposure time takes part in the number of photons collected by the detector, as well as in the contrast of the speckle pattern. It is important to mention that the information obtained by PPG and SPG shows a parabolic function with different constants on the sensitivity versus integration time. Integration time improves the sensitivity to PPG up to a certain point; then, it starts decreasing due to the averaging in time of heart-cycle information. Therefore, volumetric flow has an impact on the optimum exposure time. A similar mechanism occurs with the sensitivity to speckle contrast [17], but we cannot assume that the optimum exposure time for both PPG and SPG coincides.

The idea behind this experiment was to experimentally find an optimum integration time for simultaneous PPG and SPG obtained from single exposure videos, which maximized the heart-beat information for humans in comparison with the noise. As suggested by Yuan et al., “the measured sensitivity averaged over all animals as a function of camera exposure time and illustrates that the lowest sensitivity is observed at exposure times less than approximately 2 ms. Above this value, however, the sensitivity remains approximately constant” [18]. However, we cannot assume the same for humans considering the significant differences in volumetric blood flow, blood pressure, and differences in anatomy. In other studies, Renzhe Bi et al. used an exposure time of 2 ms [19]. K. Murali et al. defined the relationship of speckle contrast versus exposure time (T), with T ranging from 0.05 ms to 2 ms, in an intralipid phantom showing a 60% decrease in the speckle contrast at 0.5 ms [15].

In our system, the amount of information of the SPG primary peak and harmonics in comparison to the noise increased slightly, from 0.8 ms until 1.2 ms; then, it stabilized. More experiments are recommended, with a broader range of subjects performed and a kind of optical arrangement to determine the optimum exposure time for a bigger population with a varying range of volumetric blood flow values.

## 6. Conclusions

In contrast to rPPG, rSPG signal quality seems to profit from reflection light-paths. Transmission measurements seem more challenging for rSPG technology. This is likely due to the loss of coherence and mixing with intensity-induced variations. Additionally, in contrast to rPPG, rSPG signal quality does not benefit from the increase in integration times in a significant way.

In transmission measurements, coherent and non-coherent light produce nearly identical waveforms for rSPG and rPPG signals. ITA was designed as an alternative method, based on the spatial distribution of intensity, and indeed, produces very similar waveforms as rPPG. However, the ITA waveforms also are just as similar to transmissive mode rSPG. Thus, the ITA experiment demonstrated rSPG crosstalk with intensity-induced signals during transmissive measurements and provides an explanation of the appearance of rSPG signals with non-coherent light when processing with the rSPG method. Coherence appears to be lost and spatial variability signals are ruled by ITA; thus, the applied spatial rSPG algorithm, in fact, produces rPPG signals. The results raise a concern for other transmissive systems, because even when obtaining a speckle pattern, they might also be (at least partially) suffering from this effect (and thus might contain both volumetric information mixed in the SPG).

In reflection measurements, the separation between rPPG and rSPG is significant, with rSPG clearly originating from changes in speckle pattern. It looks as though reflective mode rSPG is ruled by a different physiological mechanism from volumetric pulsation, making rSPG and rPPG signals fundamentally different. Previous studies suggest that rSPG is purely flowmetric [6], but this was based upon experiments outside human physiology in which only the flow was varied. We will conduct further investigations with the intention to shed more light on the topic.

## Figures and Tables

**Table 1:** Experimental modes and their illumination settings for the 4 video channels.

MODES	LIGHT 1	LIGHT 2	LIGHT 3	LIGHT 4
**1. REFLECTIVE**	639 nm Laser Reflection	850 nm Laser Reflection	639 nm LED Reflection	850 nm LED Reflection
**2. TRANSMISSIVE**	639 nm Laser Transmission	850 nm Laser Transmission	639 nm LED Transmission	850 nm LED Transmission
**3. COHERENT**	639 nm Laser Reflection	850 nm Laser Reflection	639 nm Laser Transmission	850 nm Laser Transmission
**4. NON-COHERENT**	639 nm LED Reflection	850 nm LED Reflection	639 nm LED Transmission	850 nm LED Transmission

**Table 2:** Summary of signal quality findings on PPG. Green represents a high frequency density of PPG primary peak and harmonics compared with the system noise (SQI average > 0.85). Red represents a low frequency density of PPG primary peak and harmonics compared with the system noise (SQI average < 0.85).

PPG QUALITY	LIGHT 1	LIGHT 2	LIGHT 3	LIGHT 4
**1. REFLECTIVE**	639 nm Laser Reflection	850 nm Laser Reflection	639 nm LED Reflection	850 nm LED Reflection
**2. TRANSMISSIVE**	639 nm Laser Transmission	850 nm Laser Transmission	639 nm LED Transmission	850 nm LED Transmission
**3. COHERENT**	639 nm Laser Reflection	850 nm Laser Reflection	639 nm Laser Transmission	850 nm Laser Transmission
**4. NON-COHERENT**	639 nm LED Reflection	850 nm LED Reflection	639 nm LED Transmission	850 nm LED Transmission

**Table 3:** Summary of signal quality findings on SPG. Green represents a high frequency density of the PPG primary peak and harmonics compared with the system noise (SQI average > 0.85). Red represents a low frequency density of the PPG primary peak and harmonics compared with the system noise (SQI average < 0.85).

SPG QUALITY	LIGHT 1	LIGHT 2	LIGHT 3	LIGHT 4
**1. REFLECTIVE**	639 nm Laser Reflection	850 nm Laser Reflection	639 nm LED Reflection	850 nm LED Reflection
**2. TRANSMISSIVE**	639 nm Laser Transmission	850 nm Laser Transmission	639 nm LED Transmission	850 nm LED Transmission
**3. COHERENT**	639 nm Laser Reflection	850 nm Laser Reflection	639 nm Laser Transmission	850 nm Laser Transmission
**4. NON-COHERENT**	639 nm LED Reflection	850 nm LED Reflection	639 nm LED Transmission	850 nm LED Transmission

**Table 4:** Linear regression statistics of rPPG vs. rSPG data with a 95% prediction interval.

Regression Statistics rPPG versus rSPG	
R-Squared	0.997
Standard Error	1.778
Observations	10,058

**Table 5:** Linear regression statistics of rPPG vs. ITA data with a 95% prediction interval.

Regression Statistics rPPG versus ITA	
R-Squared	0.973
Standard Error	5.229
Observations	10,058

## Data Availability

The data presented in this study are not publicly available due to privacy related to the inclusion of human subjects.

## Supplementary Materials

The following supporting information can be downloaded at: https://www.mdpi.com/article/10.3390/s22166059/s1.
