# Supplementary material for: Camera-Derived Photoplethysmography (rPPG) and Speckle Plethysmography (rSPG): Comparing Reflective and Transmissive Mode at Various Integration Times Using LEDs and Lasers
Source: Sensors (Basel). 2022 Aug 13;22(16):6059. doi: 10.3390/s22166059 (PMC9412985; doi:10.3390/s22166059)
Supplement: Supplementary file 1 [file sensors-22-06059-s001.zip › sensors-1822064-supplementary.pdf]

We applied different signal analysis pipelines to the same raw video sequences. As for the choice of integration time, this implied that we needed to find a setting that offered both good SPG and PPG results, preferably with as little as possible low-quality outliers. When evaluating SQI boxplots for various integration times, we noticed that overall, when going beyond 1200us, the chance of getting low quality outliers clearly rose, whereas for some volunteers the signal rose only marginally or insignificantly.

Here we present a subsection showing all integration times. Each figure shows 4 PPG and 4 SPG synchronous signals from a random volunteer, each figure at different integration times. We decided to show transmission and reflection measurements, which already contain coherent and incoherent signals to avoid repetition.

We agree with the reviewer, that this information might be interesting for some specialized readers, but in order to not disrupt the reading line of the overall article, we suggest providing with this information as supplementary material.

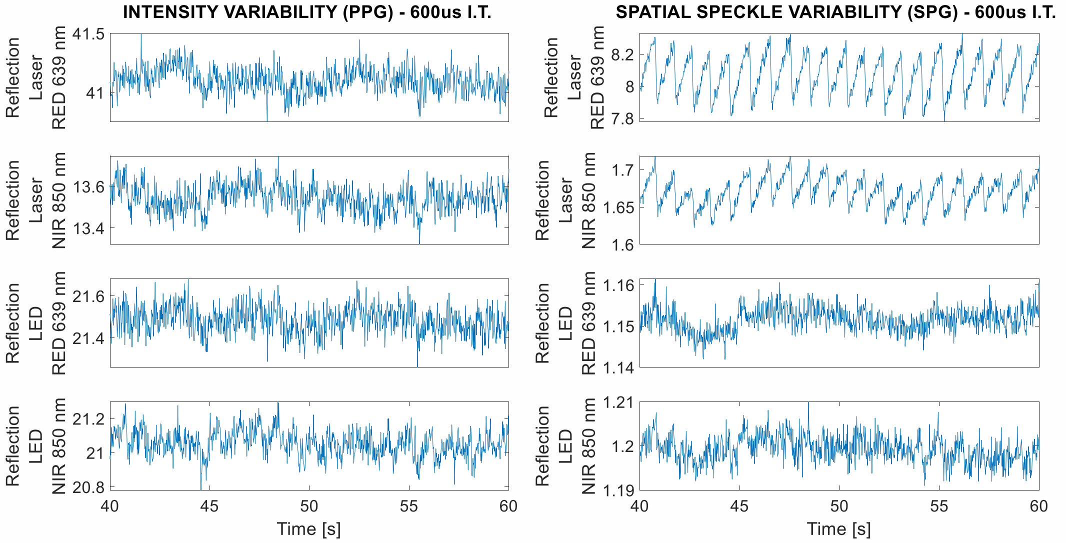

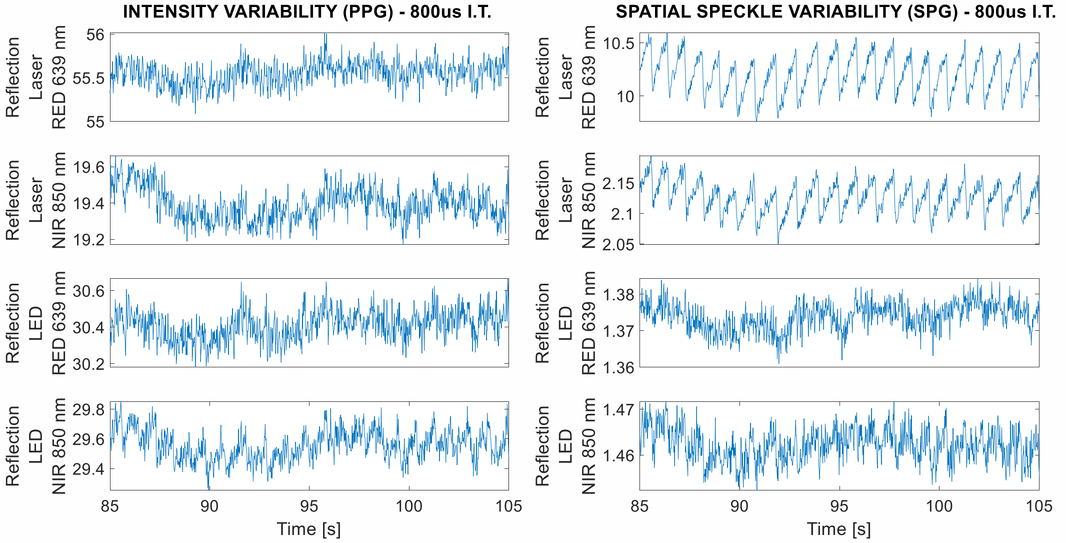

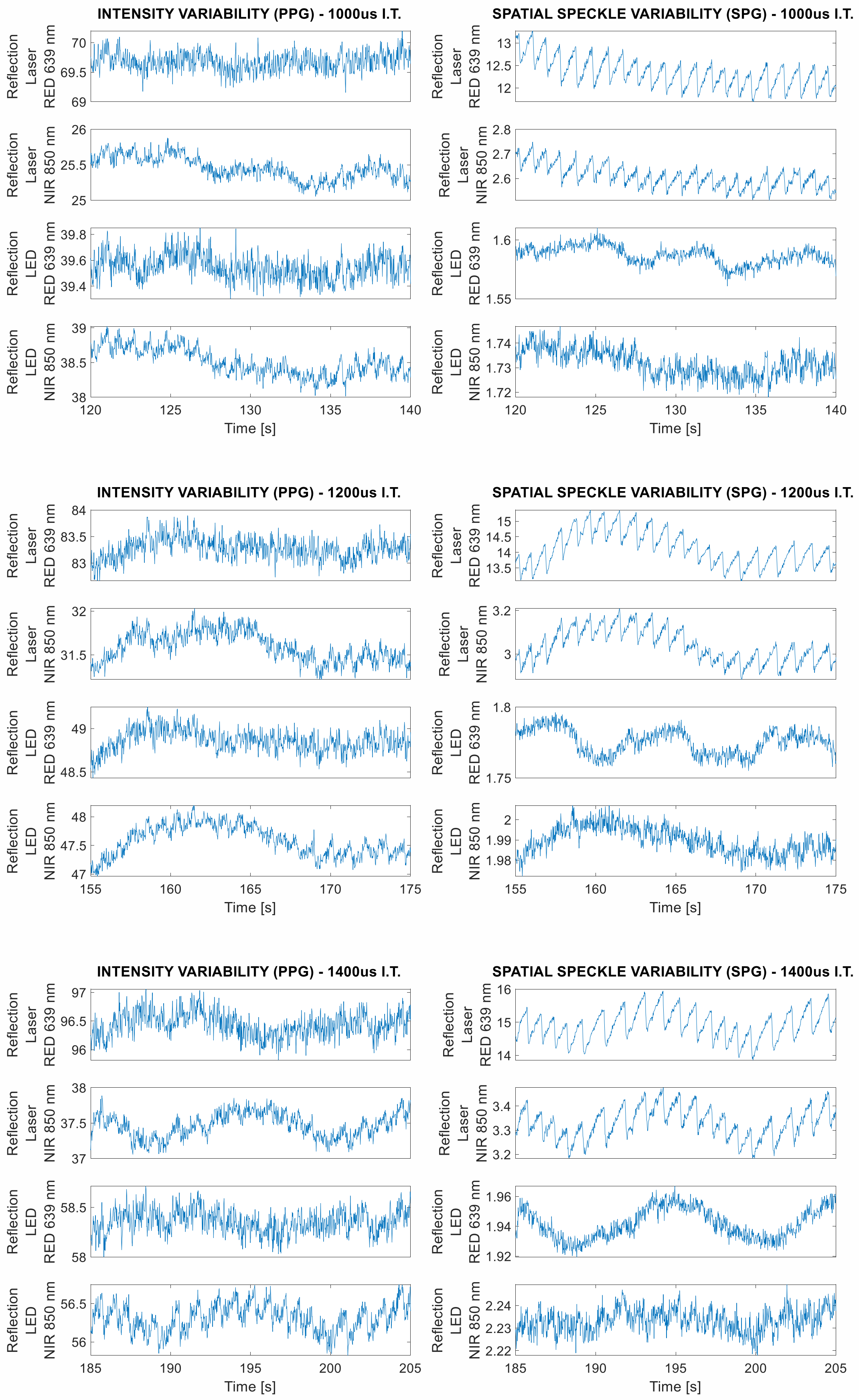

INTENSITY VARIABILITY (PPG) - 1000us I.T.
SPATIAL SPECKLE VARIABILITY (SPG) - 1000us I.T.
INTENSITY VARIABILITY (PPG) - 1200us I.T.
SPATIAL SPECKLE VARIABILITY (SPG) - 1200us I.T.
INTENSITY VARIABILITY (PPG) - 1400us I.T.
SPATIAL SPECKLE VARIABILITY (SPG) - 1400us I.T.
Reflection Laser RED 639 nm
Reflection Laser NIR 850 nm
Reflection LED RED 639 nm
Reflection LED NIR 850 nm
Time [s]

TRANSMISSION SIGNALS

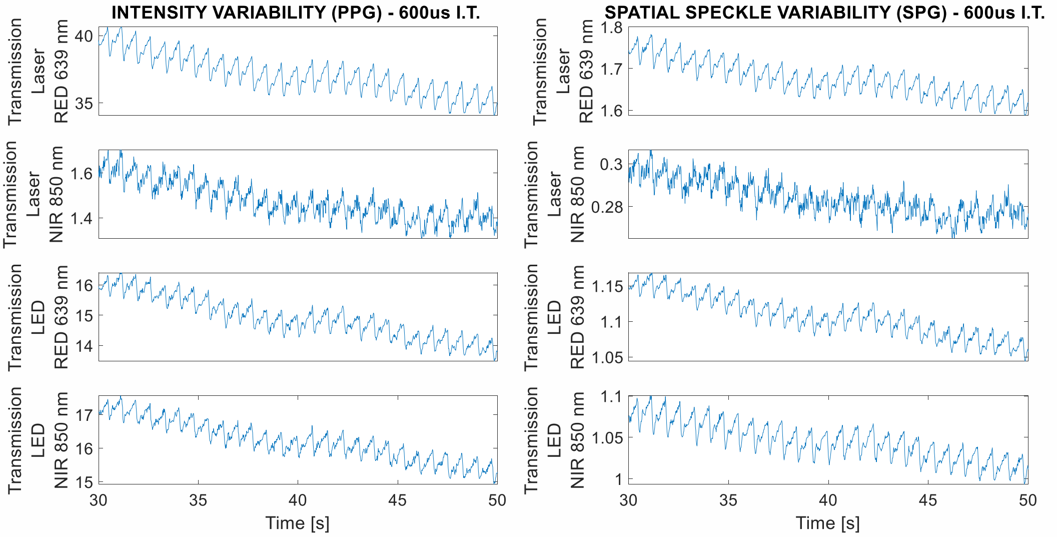

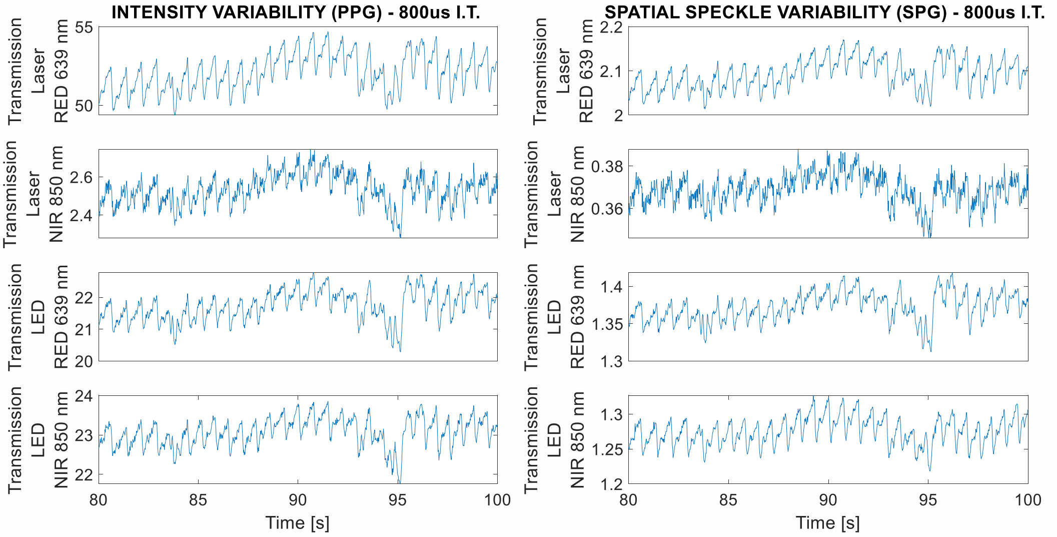

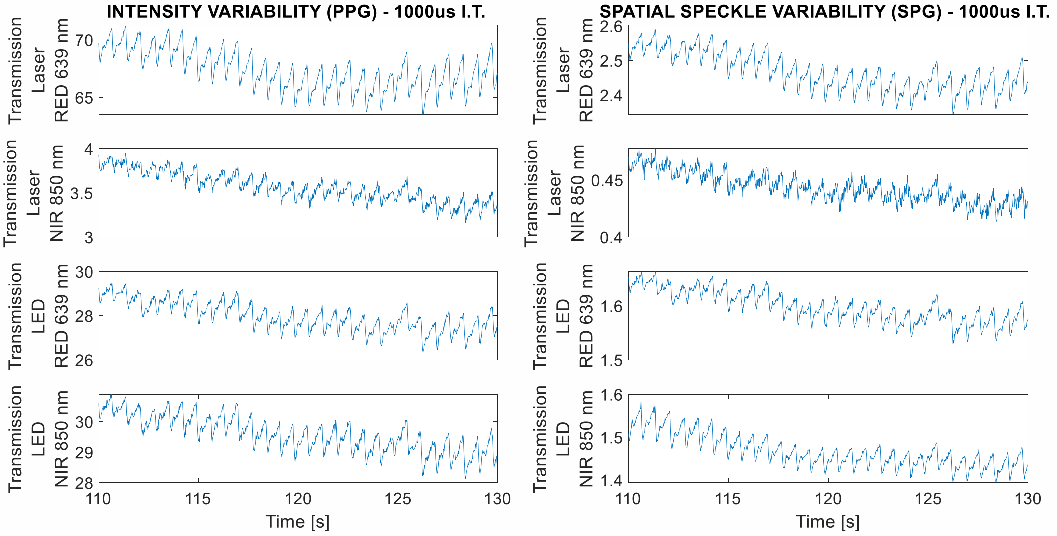

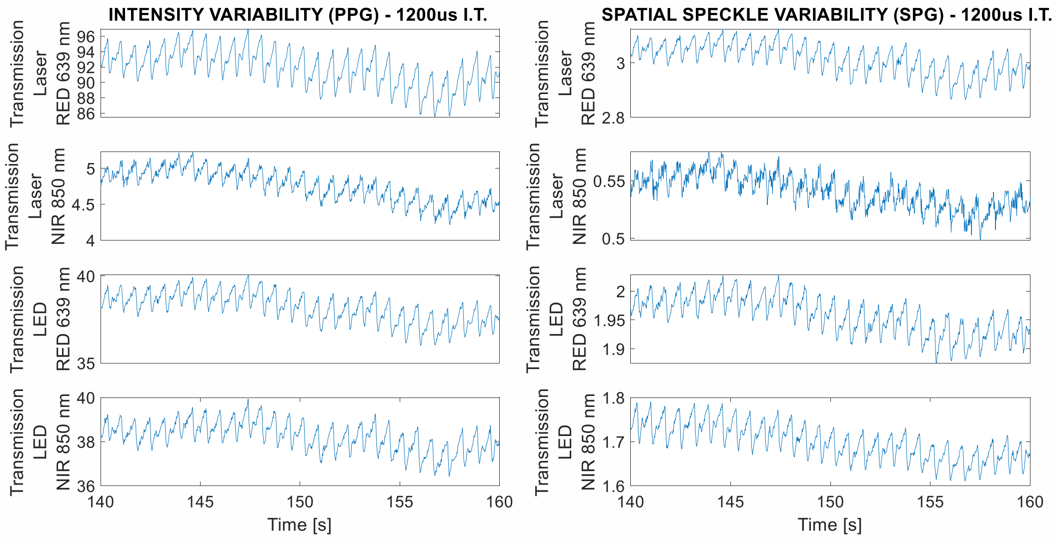
INTENSITY VARIABILITY (PPG) - 1200us I.T.
SPATIAL SPECKLE VARIABILITY (SPG) - 1200us I.T.
Transmission Laser RED 639 nm
Transmission Laser NIR 850 nm
Transmission LED RED 639 nm
Transmission LED NIR 850 nm
96
94
92
90
88
86
5
4.5
4
40
35
40
38
36
3
2.8
0.55
0.5
2
1.95
1.9
1.8
1.7
1.6
140
145
150
155
160
Time [s]

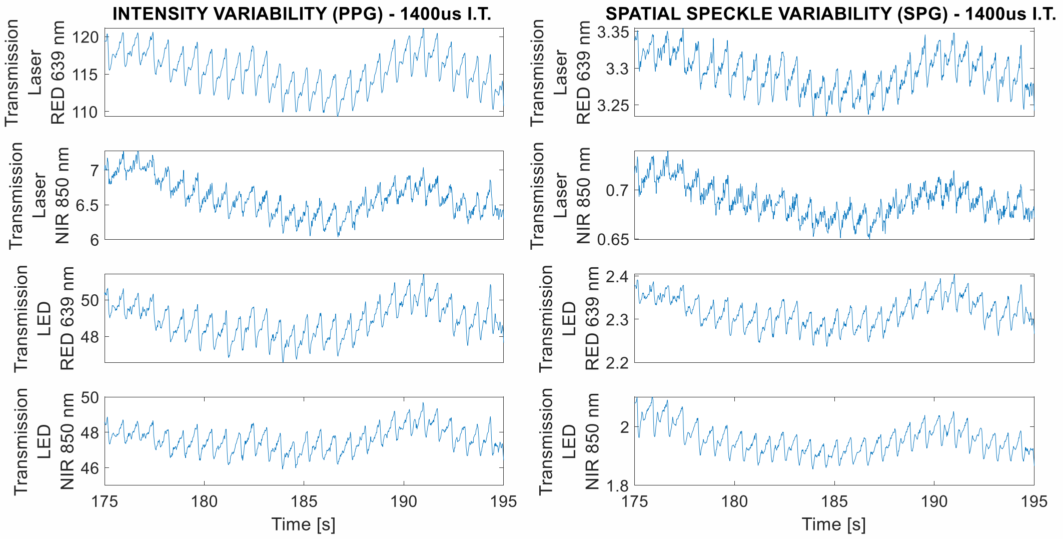
INTENSITY VARIABILITY (PPG) - 1400us I.T.
SPATIAL SPECKLE VARIABILITY (SPG) - 1400us I.T.
Transmission Laser RED 639 nm
Transmission Laser NIR 850 nm
Transmission LED RED 639 nm
Transmission LED NIR 850 nm
120
115
110
7
6.5
6
50
48
50
48
46
3.35
3.3
3.25
0.7
0.65
2.4
2.3
2.2
2
1.8
175
180
185
190
195
Time [s]
